# Exploring factors affecting the acceptance of fall detection technology among older adults and their families: a content analysis

**DOI:** 10.1186/s12877-024-05262-0

**Published:** 2024-08-20

**Authors:** Hsin-Hsiung Huang, Ming-Hao Chang, Peng-Ting Chen, Chih-Lung Lin, Pi-Shan Sung, Chien-Hsu Chen, Sheng-Yu Fan

**Affiliations:** 1 Department of Biomedical Engineering, National Cheng Kung University, Tainan, Taiwan, ROC; 2 Medical Device Innovation Center, National Cheng Kung University, No.138, Shengli Rd., North District, Tainan City, 704 Taiwan, ROC; 3 Department of Electrical Engineering, National Cheng Kung University, Tainan, Taiwan, ROC; 4 Department of Neurology, National Cheng Kung University Hospital, Tainan, Taiwan, ROC; 5 Department of Industrial Design, National Cheng Kung University, Tainan, Taiwan, ROC; 6 Institute of Gerontology, National Cheng Kung University, Tainan, Taiwan, ROC

**Keywords:** Fall, Information and Communications Technology (ICT), Innovation resistance, Content analysis

## Abstract

**Background:**

This study conducted in-depth interviews to explore the factors that influence the adoption of fall detection technology among older adults and their families, providing a valuable evaluation framework for healthcare providers in the field of fall detection, with the ultimate goal of assisting older adults immediately and effectively when falls occur.

**Methods:**

The method employed a qualitative approach, utilizing semi-structured interviews with 30 older adults and 29 families, focusing on their perspectives and expectations of fall detection technology. Purposive sampling ensured representation from older adults with conditions such as Parkinson's, dementia, and stroke.

**Results:**

The results reveal key considerations influencing the adoption of fall-detection devices, including health factors, reliance on human care, personal comfort, awareness of market alternatives, attitude towards technology, financial concerns, and expectations for fall detection technology.

**Conclusions:**

This study identifies seven key factors influencing the adoption of fall detection technology among older adults and their families. The conclusion highlights the need to address these factors to encourage adoption, advocating for user-centered, safe, and affordable technology. This research provides valuable insights for the development of fall detection technology, aiming to enhance the safety of older adults and reduce the caregiving burden.

**Supplementary Information:**

The online version contains supplementary material available at 10.1186/s12877-024-05262-0.

## Introduction

As the population of older adults grows, an emerging concern revolves around the prevalence of falls. Age-related gait and balance issues are prevalent and significant in the older adults, increasing the risk of falls and injuries [1]. Falls can result in a range of injuries, such as fracture or head injury [2, 3]. Undoubtedly, the aging population faces a substantial risk related to falls, leading to both mortality and morbidity [4]. In the United States, statistics indicate that in 2018, 27.5% of adults aged 65 and older reported experiencing at least one fall in the previous year [5]. One out of five falls results in severe injuries, such as fractures or head trauma. These falls incurred a staggering $50 billion in total medical expenses in the US in 2015 [6]. There has been a concerning rise in the number of falls resulting in injuries over the years. One study revealed that only 39% of older individuals reported experiencing a fall [7]. Furthermore, research suggests that the impact of falls continues to affect both admitted and non-admitted older adults, leading to a reduced quality of life for up to nine months following the injury [8]. On the one hand, a study revealed significant concern and fear among individuals regarding the possibility of the older adults experiencing another fall [9]. On the other hand, time on the ground (TOG) has been identified as a crucial factor affecting prognosis after a fall. TOG refers to the duration an individual remains on the ground after falling. This factor has been specifically examined in dementia patients, as falls frequently occur in memory care facilities [10]. However, falls occurring within the home environment during old age often signal the presence of severe underlying health conditions, especially without intime assistance like memory care facilities [11]. Obviously, falls among older adults is an imperative issue that needs to be addressed.

Given the fact that falls pose a significant concern in healthcare and for family caregivers, there is a growing interest in the development of methods to detect falls. Previous studies on fall detection technology explore the use of sensors in detecting fall-related events among older individuals [12–14]. One study states that fall detection technology covers three dimensions, including wearable devices, camera-based devices, and ambiance devices. It's worth mentioning that many fall detection methods are already mature and commercially available. These include video-based systems using cameras to monitor movements, microwave-based methods with radar technology to detect falls, and acoustic monitoring that analyzes sounds to identify fall events. These technologies provide valuable alternatives and enhancements to sensor-based fall detection systems [15]. Wearable devices gather data on body posture and movement, utilizing algorithms to determine if a fall has occurred. Cameras strategically positioned enable ongoing monitoring of older adults, with captured data stored for subsequent analysis and reference. Ambience devices are placed in the surroundings, like walls, floors, and beds. Data from sensors are collected, and an algorithm analyzes the input to determine if a fall has occurred [14]. Another study found that many solutions also use mobile device sensors, particularly accelerometers, for fall detection in older adults [13]. The above literature review provides examples of fall detection technology application areas that already exist in the market. Therefore, fall detection technology among older adults has the potential to alleviate the societal burden. However, technology-based solutions, despite their potential benefits, often face resistance from older adults, creating barriers to the adoption of health-related information and communication technology. To address these barriers, we conducted a comprehensive literature review, examining the challenges that older adults may encounter when using fall detection technology.

In 1987, Ram introduced an innovation resistance model [16], aiming to address the reluctance of consumers to adopt new innovations, particularly when these innovations have the potential to disrupt their existing satisfaction levels or clash with their established beliefs. Building upon this framework, Ram and Sheth [17] (1989) identified a range of obstacles that hinder consumers' willingness to embrace innovations, classifying them into two main categories: functional barriers and psychological barriers. Functional barriers encompass aspects such as usage limitations, value considerations, and risk perceptions. We conducted a literature review on the barriers that older adults may face when using the technology. Among usage barriers, age-related factors, including hearing impairments, reduced dexterity, declining vision, and mild cognitive challenges, can significantly impact the ease with which users adopt new technologies [18–22]. Previous research [18, 23–26] has emphasized that technical unfamiliarity, which includes inadequate technical skills, a lack of understanding about how to use technology, and limited computer literacy, poses significant challenges for older individuals in adopting new technologies. Additionally, a lack of clear and comprehensive instructions has been identified as a common obstacle for older adults in the literature [24, 27, 28]. Given that the value barrier concept suggests innovative products must offer greater value than existing ones to motivate consumers to switch, there is a scarcity of references related to this description. On the other hand, risk barriers encompass concerns about product reliability, including issues like false alarms and inaccurate data, which can be functional risks that older individuals may encounter [19, 27, 29–31]. High costs also contribute to risk barriers. Many older adults are concerned about the price of the product itself [22, 30, 32]. Furthermore, privacy concerns have been raised by many older individuals, adding to the array of issues related to risk barriers [18, 21, 22, 33, 34].

Psychological barriers encompass traditional belief barriers and image-related barriers. Older adults also encounter psychological barriers when using information and communication technology. Among older adults, attitude toward technology represents a common traditional belief barrier, reflecting issues related to trust in their ability to manage devices and their reluctance to adopt it [18, 21, 35]. Image barriers involve concerns about a product's appearance [27], with some older individuals perceiving certain products as designed for younger generations, which may deter their adoption [24].

While numerous articles have explored the barriers older individuals face in adopting information and communication technology (ICT) [18, 22, 36], it's essential to acknowledge that ICT encompasses a wide range of applications, making it a diverse and multifaceted topic. Within healthcare, various applications exist, which can make it challenging for healthcare providers to develop products that cater specifically to their target users. While the previous studies encompass fall prevalence, economic burden of falls, and the challenges older adults may face when using ICT, this study focuses more on barriers of these technological products used by older adults and their families, providing a valuable evaluation framework that can aid healthcare providers, particularly in the field of fall detection. Through this research, we aim to offer a valuable assessment framework for making the best use of ICT to help older adults immediately and effectively when falls happen.

## Methods

### Study design

In order to address our research inquiry on the perceived challenges associated with the adoption of fall detection technology and expectations of fall detection technology among older adults and their families, we employed a qualitative approach. Our primary sources of data analysis were semi-structured interviews from in-depth interviews. In-depth interviews are widely acknowledged and commonly used in qualitative research [37]. The semi-structured interview outline utilized in our study provided a well-defined yet flexible and open-ended framework for exploring the topic [38]. To align with the research objectives, we developed a semi-structured interview outline, including the background of participants, expectations of fall detection technology, and innovation resistance (see Tables 1 and 2). Face-to-face interviews were then conducted with older adults along with their families.

**Table 1:** Outline of semi-structured interview on factors influencing the acceptance of fall detection technology (older adults)

1. What is your current health condition? Are there any discomforts or troubles in your daily life? Do you face any challenges or inconveniences in walking or moving? How is your balance ability?
2. Have you experienced any falls before? If yes, where did these incidents occur, and what were the circumstances? Can you describe your most recent fall? What were the conditions surrounding it?
3. Have you had multiple falls? If so, were there any differences or specific aspects in these experiences? What concerns do you have regarding falling?
4. In your opinion, what situations are more likely to lead to falls? This includes specific contexts and how these falls typically happen. What impact and inconveniences, both physical and psychological, result from falls?
5. If you haven't experienced a fall, considering your balance and mobility, do you still worry about falling? What aspects concern you, and why?
6. How is your current living environment? Where do you spend most of your time at home? And outside, where do you usually go? Are there any inconveniences or dangers in these places? How do these environments affect your life?
7. How do you manage the risk of falling? Are there any methods or adjustments you make? Regarding fall detection technology, what expectations or desires do you have for such products? What functionalities or effects do you hope they possess? Would you be willing to use them?
8. Do you think the fall detection technology would be helpful for older individuals? What functionalities do you expect this system to have? What appearance/design/features would you expect it to have? What benefits do you anticipate it providing? What problems could it potentially solve?
9. What challenges do you foresee in using a system like this? How would you evaluate its usefulness? What key indicators would you use to assess its ease of use?
10. When evaluating this type of product, what risks would you consider? What concerns or apprehensions would you have?
11. How do you think this product differs from traditional methods used for fall prevention? Which differences might affect your willingness to use it?
12. Do you think using this product would have any impact on your self-image? If yes, what kind of impact would it have?

**Table 2:** Outline of semi-structured interview on factors influencing the acceptance of fall detection technology (family)

1. What care do older individuals at home require? What responsibilities do you have in caring for them?
2. How is the walking and balance ability of older adults? Have they experienced any falls?
3. If they have fallen, what were the circumstances? What impact did it have on older adults? How about the impact on you or your family?
4. If they haven't fallen, do they worry about the risk of falling? Do you worry about it?
5. In the current living environment, what are the risk factors for falls? Have any adjustments or fall prevention mechanisms been implemented?
6. If there were technological products for preventing falls designed for older adults at home, what would be your willingness and considerations for them to use?
7. Do you think the fall detection technology would be helpful for older individuals? What functionalities do you expect this system to have? What appearance/design/features would you expect it to have? What benefits do you anticipate it providing? What problems could it potentially solve?
8. What issues do you foresee regarding the use of this type of system? How would you evaluate its usefulness? What key indicators would you use to assess its ease of use?
9. What problems do you think this product might encounter in use? What indicators do you think can evaluate its ease of use?
10. When evaluating this type of product, what risks would you consider? What concerns would you have?
11. In your opinion, what are the differences between this type of product and traditional methods used for fall prevention? Which differences might affect your willingness to purchase and use it?
12. Do you think the user might feel that this type of product is unattractive or be concerned about being laughed at by others? How do you think it might affect their self-perception and yours?

### Study subject and recruitment

The aim of this study was to understand the perspectives of older adults with chronic disease, who are prone to falls [1], and their family caregivers, who are the older adults’ spouses or children. Purposive sampling was employed, and specific inclusion criteria were set for the study participants. These criteria consisted of: (1) healthy individuals over the age of 20 who agreed to participate; (2) participants aged 45 or above, including those affected by stroke, frailty, dementia, Parkinson's disease, and other diseases; (3) participants whose condition was stable, able to mobilize, and willing to take part in the study. We included participants younger than 60 years old in our study because they have chronic diseases such as stroke, dementia, and Parkinson's disease. Individuals with these conditions are more prone to falls compared to others. Although these diseases are typically associated with older adults, we believe that younger participants with these conditions are potential future users of fall detection technology. Therefore, our sample includes individuals under 60 years old and their respective family caregivers.

To ensure clear comprehension of the study's purpose, procedures, and potential risks, an individualized approach was adopted in explaining the study to each participant. Additionally, oral explanations were provided to ensure their understanding of the research instructions and terms outlined in the consent form. In total, interviews were conducted with 30 older adults and 29 families (with one family unable to attend).

### Data collection

The study received ethical approval from the Human Research Ethics Review Committee, and the case number assigned was A-ER-110–211. From September 2022 to April 2023, in-depth interviews were conducted in NCKU outpatient hospital using a semi-structured interview outline. The interview process began with the researchers introducing themselves to the participants and providing a detailed explanation of the study's purpose, the interview procedure, and the rights of the participants. Privacy regulations were emphasized, assuring the interviewees that their personal data would be treated confidentially. Following comprehension of the study's objectives and their rights, the participants were informed about the recording of the interview. It was made clear that if they preferred not to be recorded, the investigators would respect their decision and take handwritten notes instead. Each interview lasted approximately 40–60 min. After each interview, research assistants were responsible for transcribing the recorded interview files to create a written transcript of the data. Prior to analysis, the researchers reviewed the verbatim transcripts of the interviews to ensure accuracy and identify any potential errors. If any inconsistencies or missing information were found, another researcher would review the audio recording and the transcript to ensure accuracy and correct any deviations from the original intended meaning.

### Data analysis

The qualitative interview data in this study was subjected to content analysis. To streamline the content analysis process and identify themes within the qualitative responses, a panel consisting of four members was established. In addition, the whole process of data analysis was supervised by the professor. The panels include one doctoral researcher, one research assistant, and two graduate students. In employing the inductive approach, 4 researchers employed a systematic process that involved dividing the data into distinct units of meaning, condensing these units, assigning codes, categorizing the codes, and identifying overarching themes [39, 40]. The analysis began with the researchers thoroughly reading and rereading the interview data, treating each segment as a unit of analysis. Similar statements within the text were identified and extracted to form meaning units. These meaning units were then condensed through a careful reduction process while ensuring the preservation of their core essence. Subsequently, the meaning units were systematically coded based on their content, with researchers assigning specific codes to each unit. Once the coding process was complete, all the codes were further organized into meaningful categories. Finally, the researchers identified and grouped together different categories that shared related underlying meanings, thereby forming overarching themes [41]. This rigorous approach to content analysis enabled a comprehensive exploration and interpretation of the qualitative interview data in the study.

## Results

### Respondent characteristics

From September 2022 to April 2023, the study included 30 older adults and 29 family members, all recruited from NCKU Medical Center in Taiwan. These participants are referred to as N_ Interviewee (older adults /family). The older adults, primarily diagnosed with Parkinson's disease, dementia, or stroke, were selected based on their scores on the Morse Scale [42], Clinical Frailty Scales [43], and Barthel Index [44]. Additionally, the study documented the history of fall events and the relationship between the older adults and their family. Among the older adults, 19 older adults had experience using smartphones, while the remaining older adults did not have the experience (Table 3).

**Table 3:** Personal characteristics of older adults with chronic disease

NO	Gender	Age (years)	MORSE*	Clinical frailty scales	Barthel index	History of falls one year ago	Diagnosis	Using smartphone experience	Family relationship
1	Female	70	2(50)	6	75	Yes	Dementia	Yes	Couple
2	Male	73	1(15)	4	90	No	Dementia	No	Daughter
3	Female	52	1(0)	3	95	Yes	Stroke	Yes	Couple
4	Male	68	2(40)	2	100	Yes	Stroke	Yes	Couple
5	Female	84	1(0)	4	85	Yes	Dementia	No	Son
6	Male	63	1(0)	1	100	Yes	Stroke	Yes	Couple
7	Female	61	1(0)	1	100	Yes	Chronic dizziness	Yes	Daughter
8	Male	56	2(25)	2	100	No	Dementia	Yes	Couple
9	Female	81	2(25)	4	95	Yes	Dementia	No	Couple
10	Female	74	1(0)	2	100	Yes	Parkinson	Yes	Daughter
11	Female	71	1(0)	2	100	Yes	Parkinson	Yes	Daughter
12	Male	83	2(25)	3	95	Yes	Stroke	No	Son & Daughter
13	Male	83	2(30)	2	100	No	Stroke	Yes	Couple
14	Male	87	1(10)	4	95	No	Dementia	No	Couple
15	Male	78	1(0)	2	100	No	Stroke	No	Couple
16	Female	75	1(15)	6	75	Yes	Dementia	No	Daughter
17	Female	69	1(0)	1	100	Yes	MCI*	Yes	Couple
18	Male	70	1(0)	1	100	Yes	Joint problems	Yes	Couple
19	Female	65	1(0)	1	100	Yes	HTN*	Yes	Couple
20	Male	70	1(0)	1	100	No	HTN	Yes	Couple
21	Female	63	1(15)	6	55	No	Dementia	No	Daughter
22	Male	64	1(0)	1	100	No	Stroke	Yes	Couple
23	Female	64	1(0)	1	100	No	Stroke	Yes	Daughter
24	Male	85	1(0)	2	100	Yes	Dementia	No	N/A
25	Female	86	2(30)	4	95	Yes	MCI	No	Daughter
26	Male	74	1(0)	2	100	Yes	Parkinson	Yes	Couple
27	Male	69	2(40)	6	50	Yes	Parkinson	No	Couple
28	Male	65	3(55)	3	90	Yes	Parkinson	Yes	Daughter
29	Male	88	2(40)	4	100	Yes	Dementia, Parkinson	Yes	Son
30	Male	75	2(40)	6	65	Yes	Dementia, Parkinson	Yes	Couple

^*^Morse: 1. No risk: 0 ~ 24; 2. low risk: 25 ~ 50; 3. high risk: >  = 51

^*^MCI Mild Cognitive Impairment, *HTN* Hypertension

### Theme

**Fig. 1:**
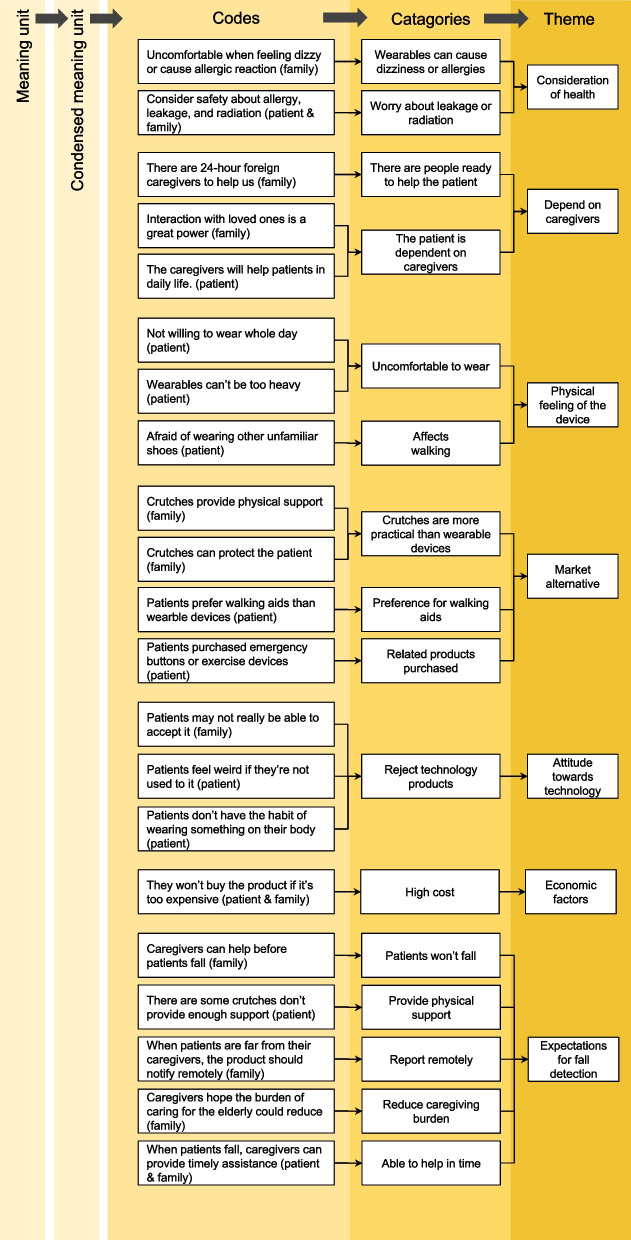
Factors influencing adoption of fall detection technology in older adults and families

Based on the interviews conducted with older adults and their families, we have identified the primary considerations influencing the decision to use wearable fall-detection devices (as detailed in Fig. 1; Appendix). These considerations span various aspects, including (1) health considerations, (2) reliance on human care, (3) personal comfort issues, (4) market alternatives, (5) attitude towards technology, (6) financial concerns, and (7) expectations for fall detection technology. The main factors are described below.

### Health considerations

Concerns about potential health risks associated with wearable fall-detection devices emerged as a significant barrier to their adoption. older adults and their families expressed apprehensions about adverse effects such as dizziness, skin irritation, electrical leakage, and electromagnetic radiation. These concerns are particularly pronounced among older individuals, who tend to be more cautious about new technologies that interact directly with their bodies.

*“Yeah, older adults won’t wear it if it's uncomfortable; it's just about avoiding dizziness.” (8_family)*

For instance, some family members voiced worries about the possible radiation-related functions of these devices. Others were concerned about the risk of skin allergies and electrical leakage due to the close contact of these devices with the skin. These apprehensions highlight a broader fear of unknown health impacts, which can deter older adults from embracing new technological solutions for fall detection.

*“Well, just now, it's just that I've heard that there might be some concerns about it. Because it's worn on the skin, so there's a fear of it having some impact on their skin. Also, there's the question of whether it might have electrical leakage.” (6_family)*

*“Perhaps, he has some kind of fear, like he might think that this thing could cause harm to the body? Or maybe he's worried about things like skin allergies or getting an electric shock, and so on.” (20_family)*

### Reliance on human care

Despite the potential benefits of fall-detection technology, many participants in the study emphasized a strong preference for human care and assistance. The majority believe that hiring caregivers or relying on family members is a more reliable and comforting approach. This trust in human assistance is deeply rooted and may significantly hinder the adoption of technological solutions.

Several older adults indicated that they felt no need for fall-detection devices because they were constantly accompanied by attentive family members or professional caregivers. For instance, some older adults mentioned that their spouses or foreign domestic workers were always available to assist them with daily activities, rendering the technology unnecessary. Others noted that their children, who are medical professionals, provided adequate care, further diminishing the perceived need for such devices.

Additionally, the cultural context plays a significant role in this reliance on human care. The close-knit family structure and the high value placed on personal interaction and caregiving contribute to the resistance against technological interventions. Many participants expressed a preference for investing in human care over spending money on devices, indicating that they view personal care as more effective and compassionate.

*“Most people now hire foreign domestic workers to provide care. If he needs to get up to go to the bathroom, he'll definitely inform the foreign caregiver, saying, "I need this, I need that, please help me up.” (22_older adults)*

*“So instead of this, we might end up hiring someone to take care of him or considering long-term care services. Because rather than spending that money, it's the same as having someone look after you 24 h a day.” (2_family)*

In summary, both health considerations and a strong reliance on human care are critical factors influencing the adoption of wearable fall-detection devices among older adults. Addressing these concerns through better education about the safety and benefits of these technologies, as well as integrating them into existing caregiving practices, may help in overcoming these barriers.

### Personal comfort issues

The comfort and practicality of wearable devices are critical concerns for potential users, significantly impacting their adoption. Key issues identified include the weight and physical discomfort of these devices. Users are generally inclined to avoid technologies that cause inconvenience or discomfort in their daily lives, highlighting the necessity for user-friendly and ergonomic designs.

Participants indicated that the weight of the devices is a primary concern; many stated a preference for lightweight options. Physical discomfort, such as restrictions in movement, emerged as a significant factor. For example, older adults expressed concerns about devices causing discomfort when attached to the knee or foot, which could interfere with their mobility and overall comfort. There is a clear preference for devices that are unobtrusive and do not hinder daily activities.

*“Fastened around the knee, I can't do it now. I'm afraid I'll get stuck when I'm walking.” (1_older adults)*

*“I care about the weight. It shouldn't be too heavy; it should be relatively lightweight.” (20_older adults)*

### Market alternatives

The preference for traditional fall prevention tools, such as canes and emergency buttons, was evident among many participants. These established solutions are familiar and trusted, making them more appealing than newer technological alternatives. Additionally, some participants believed that canes provide proactive assistance to prevent falls, whereas fall detection technology only alerts family members after a fall has occurred, which does not prevent the incident itself.

Participants noted that they already possess reliable fall prevention tools at home, such as emergency buttons, which they trust for their effectiveness in emergencies. The familiarity and simplicity of these tools make them a preferred choice over fall detection technology. Additionally, canes with stable bases are viewed as effective in ensuring personal safety and preventing falls, further reducing the perceived need for fall detection technology. To compete with traditional methods, fall-detection technology must not only match but surpass the reliability and convenience of existing tools.

*“I currently have an emergency button installed in my home. If I have an accident, I can just press that button, and the security company will come to assist me.” (19_older adults)*

*“Because he just took the crutch and walked with it. Yes, if he wears this, he will still fall.” (8_family)*

### Attitude towards technology

A prevailing theme in the interviews is resistance to change, with some older individuals expressing a reluctance to adapt to new technologies. This resistance is often rooted in perceptions of inconvenience, unfamiliarity, and a general aversion to having devices attached to their bodies. Overcoming this resistance will require addressing user concerns and providing user-friendly solutions.

Elderly individuals frequently describe new devices as uncomfortable and cumbersome. For example, one older adult noted feeling "strange" and "not used to it" when considering wearing fall-detection devices. Others expressed outright resistance, emphasizing a strong preference for maintaining their current routines without the addition of new technological elements. This sentiment is further compounded by a dislike for the perceived hassle of wearing or carrying additional items, such as glasses or wearable devices.

*“It's a strange feeling, doesn't feel like it, not used to it, feels weird.” (16_older adults)*

*“I'm just too lazy to wear glasses. We usually don't like having things hanging here and there.” (24_older adults)*

*“And to be honest, older people might have a greater psychological burden. If you ask them to carry something every day, they might not like it or feel that it restricts their mobility, and they might not want it.” (20_family)*

### Financial concerns

The cost of fall-detection devices is a significant consideration for many older adults and their families. Affordability is a key factor in their decision-making process, with financial capability greatly impacting the willingness to adopt new technology.

Many participants highlighted the financial burden that expensive fall-detection devices could impose. For families already managing substantial living expenses, the additional cost of advanced technology may be prohibitive. This financial strain is particularly acute for those on fixed incomes or with limited financial resources.

*“I don’t want this if it’s too much money.” (9_older adults)*

*“I think financial capability comes first. If there are no issues with economic conditions, you have to make sure they have the financial ability to afford it. That's the main issue.” (5_family)*

### Expectations for fall detection technology

Participants highlighted several key expectations for fall detection technology, which, if met, could facilitate its adoption. These expectations include features such as remote notifications, physical support, real-time older adults status updates, and immediate assistance functions. Meeting these expectations can enhance the perceived value of fall detection technology and increase user willingness to adopt it.

A major expectation is the ability of the technology to provide real-time notifications to caregivers or family members when a fall occurs. Participants expressed a desire for systems that could alert them regardless of their location, ensuring timely intervention. For example, one family member emphasized the need for notifications even if older adults are far away, illustrating the importance of reliable and far-reaching communication capabilities.

Another expectation is for the technology to offer some form of physical support to prevent falls before they happen. Participants envisioned devices that could sense an impending fall and provide immediate physical assistance to prevent the incident. This proactive approach would not only enhance safety but also provide peace of mind for both users and their caregivers.

Real-time older adults’ status updates and the ability to monitor the condition of older adults remotely were also highly valued. For instance, having access to visual data or images of the older adults’ home environment was seen as a way to increase the sense of security and ensure timely responses to any issues. Comprehensive data on the older adults' health and activity levels could help in managing and understanding their overall condition.

*“If we can assist her just before she falls, that would be the ideal scenario. Being able to support her right before the fall occurs.” (1_family)*

*“So, if we talk about it in terms of shoes, if it can sense that a person might slip or fall, can it prevent them from falling?” (2_family)*

*“It might be like this. If he wears it and triggers the alarm when he's far away, like what I just mentioned, if he's in Xitou and triggers the alarm, we're in Tainan.” (6_family)*

*“Data, as I just mentioned, is about being able to have a more immediate and clear understanding of the progression of the condition. And assuming that there is also the capability to capture images or, in a way, for me to see their condition at home, this might make me feel more at ease.” (10_family)*

## Discussion

The adoption of fall-detection wearable devices among older individuals and their families is influenced by a complex interplay of factors, as revealed by the findings of this study. Understanding these factors is essential for the successful integration of such technologies into the lives of older adults. The participants' concerns about safety issues, such as skin irritation, dizziness, electrical leakage and radiation, may stem from a heightened awareness of the potential risks associated with electrical products, especially for wearable devices. These concerns can deter older adults from embracing wearable information and communications technology, implying that safety issue could be the potential barrier. Similarly, another study has identified safety factors, including concerns relate to radiation and the use of electricity [45]. Thus, to address this barrier, device designers should prioritize safety issues, reducing any safety-related risks. These considerations can help alleviate concerns and enhance user’s confidence. Another theme is the preference for human care over technology, with many participants believing that caregivers or family members provided more reliable support. One review study [30] emphasizes that companionship plays a crucial role in the context of having a source of support and presence in one's life. The preference for human care in taking care of older adults suggests that fall-detection devices should be viewed as complementary tools rather than replacements for caregivers. This aligns with concerns about the fear of losing social connections and experiencing loneliness [46]. In other words, while technology can aid in ensuring safety, the emotional and social aspects provided by human caregivers are irreplaceable. This is an important finding that emphasizing this perspective may decrease the barriers of using fall detection technology among older adults.

Issues related to device comfort and practicality were highlighted as significant factors influencing adoption as well. Concerns from stakeholders include device weight and physical discomfort. Obviously, user-friendly design is essential to mitigate these concerns [47]. Designers should aim to create lightweight, comfortable devices that seamlessly integrate into daily life, or design a fall detection technology that does not require older adults to wear. In addition, participants expressed a preference for traditional fall prevention tools, such as canes or emergency buttons, citing familiarity and trust in these established solutions. Several participants voiced the opinion that a cane is more beneficial than a fall detection device since a cane can provide support to older adults and reduce the risk of falls, whereas they believe that fall detection devices may not effectively prevent older adults from falling. This concept that the product is able to prevent falls is similar to fall prediction systems [48]. On the one hand, this factor may require fall detection technology to demonstrate its superiority over existing options or complement the characteristics of existing products. On the other hand, perception of inconvenience, unfamiliarity, and embarrassment were common attitudes among older adults [19, 32, 47]. In our study, some participants also stated that fall detection devices are troublesome. We suggest making fall detection devices easy to use by designing them to be simple and not bothersome.

The cost of fall detection devices emerged as a significant consideration for both older adults and their families. Affordability is a key factor in their decision-making process [22, 27, 30, 32, 47], highlighting the importance of exploring options for making these devices more accessible, such as through insurance coverage or subsidies. On the other hand, one study investigated the preferred specifications, perceived ease of use, and perceived usefulness of an automated fall detection device among older adults who rely on wheelchairs or scooters. It was noted that participants expressed a belief in the utility and user-friendliness of an automated fall detection device. The features include wireless charging, a wristwatch-like design, the option to change the emergency contact person in case of a fall, and the ability to deactivate notifications in case of false alarms [49]. In our study, participants emphasized the importance of comprehensive fall detection solutions, including remote notifications, real-time older adults’ status updates, and immediate assistance functions. It seems that the function of fall detection technology is oriented toward notifying the families, enabling them to assist immediately. Therefore, prioritizing the creation of devices that detect falls and provide added value through additional features is beneficial for enhancing overall safety and well-being.

### Limitations

Although this study contributes to the field of fall detection technology, the study has several limitations. First, the sample of older adults comes from neurology outpatient. This limits the findings to this specific group and decreases their generalizability. Second, the findings of this study are based on the opinions and experiences of the respondents and may not be fully representative of all potential users of fall detection technology. The experiences and preferences of non-respondents remain unknown and might differ from those who participated in the study. In addition, the study involved respondents with varying levels of fall risk, as they suffered from different health conditions such as acute stroke, mild to moderate dementia, impaired cognitive function, and poor balance and gait. Third, as fall risk factors can significantly influence the perception and acceptance of fall detection technology, the results may not fully capture the nuances of specific subgroups within older population. The in-depth, face-to-face interviews were conducted in the outpatient area of the hospital. Although none of the interviewees discontinued the interviews due to privacy concerns, it is important to consider the potential influence of the interview setting. In addition, the outpatient waiting area in a hospital is an open and public space, which might have affected the responses of the interviewees. They may have been conscious of their surroundings and the presence of other individuals, possibly influencing the openness of their responses. Finally, the study focused on a specific population in Taiwan, and the findings may be influenced by cultural and regional factors unique to this context. Cultural differences and healthcare practices may lead to varying perspectives on fall detection technology in other regions or countries.

## Conclusion and suggestions

In this study, we examined the factors influencing the adoption of wearable fall-detection devices among older adults and their caregivers. We identified several key considerations: concerns about potential health risks associated with these devices, the preference for human care over technology, the importance of device comfort and practicality, market alternatives, cost considerations, the attitude towards technology, and expectations of technology. Based on our evaluation framework, it is essential to consider safety, usability, affordability, and complementary to human care when developing fall detection products. In addition, meeting user expectations for comprehensive features like remote notifications and immediate assistance functions can further enhance adoption. Addressing these factors and challenges is expected to enhance the safety and quality of life for older adults, thereby relieving the burden of care.

### Supplementary Information

Supplementary Material 1.

## Data Availability

Data is provided within the manuscript.

## Abbreviations

ICT: Information and communications technology
MCI: Mild Cognitive Impairment
HTN: Hypertension
